# mtDNA Chromatin-like Organization Is Gradually Established during Mammalian Embryogenesis

**DOI:** 10.1016/j.isci.2018.12.032

**Published:** 2019-01-08

**Authors:** Shani Marom, Amit Blumberg, Anshul Kundaje, Dan Mishmar

**Affiliations:** 1 Department of Life Sciences, Faculty of Natural Sciences, Ben-Gurion University of the Negev, Beer-Sheva 8410501, Israel; 2 Department of Genetics, Stanford University, Stanford, CA, USA; 3 Department of Computer Science, Stanford University, Stanford, CA, USA

**Keywords:** Biological Sciences, Developmental Genetics, Molecular Genetics, Developmental Biology

## Abstract

Unlike the nuclear genome, the mammalian mitochondrial genome (mtDNA) is thought to be coated solely by mitochondrial transcription factor A (TFAM), whose binding sequence preferences are debated. Therefore, higher-order mtDNA organization is considered much less regulated than both the bacterial nucleoid and the nuclear chromatin. However, our recently identified conserved DNase footprinting pattern in human mtDNA, which co-localizes with regulatory elements and responds to physiological conditions, likely reflects a structured higher-order mtDNA organization. We hypothesized that this pattern emerges during embryogenesis. To test this hypothesis, we analyzed assay for transposase-accessible chromatin sequencing (ATAC-seq) results collected during the course of mouse and human early embryogenesis. Our results reveal, for the first time, a gradual and dynamic emergence of the adult mtDNA footprinting pattern during embryogenesis of both mammals. Taken together, our findings suggest that the structured adult chromatin-like mtDNA organization is gradually formed during mammalian embryogenesis.

## Highlights

• Mouse and human mtDNA ATAC-seq footprinting patterns are formed during embryogenesis
• mtDNA footprinting sites were either occupied in preimplantation or appeared later
• mtDNA footprinting associates with regulatory elements and protein-binding sites
• The mtDNA footprinting sites tend to harbor secondary structures

## Graphical Abstract

**Figure:**
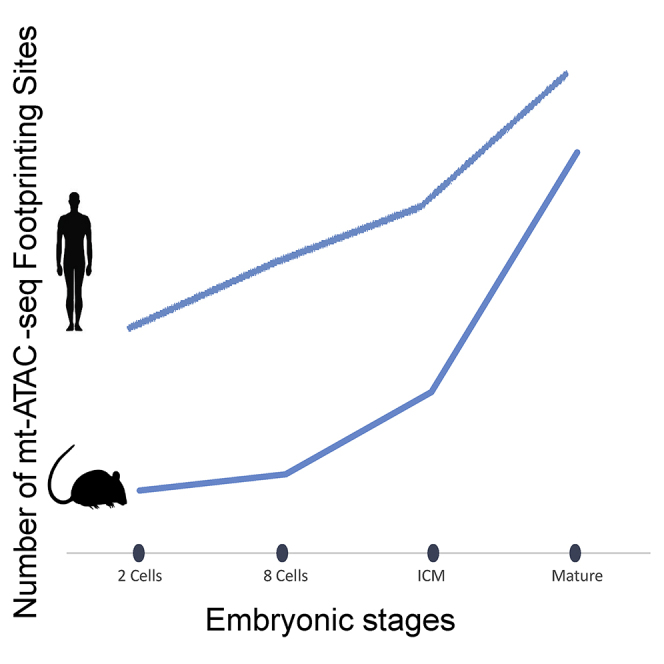

## Introduction

During metazoan embryogenesis, the transition to zygotic gene expression occurs during the blastocyst stage. This stage precedes the transformation of totipotent cells to detrimental cell fate, which was shown to depend on the metabolic switch from glycolysis to oxidative phosphorylation (OXPHOS) (reviewed by Folmes et al., 2012). As OXPHOS occurs within the mitochondria, the major player in cellular metabolism, it is not surprising that the transition toward differentiating cells is also marked by changes in mitochondrial subcellular distribution (Folmes et al., 2011), mitochondrial morphology, and numbers (Hom et al., 2011), as well as in their regulation of transcription and replication (Wellen and Thompson, 2012). Therefore, as the transition to zygotic gene expression is also accompanied by nuclear genome (nDNA) chromatin remodeling, one may expect it will also affect the higher-order organization of the mitochondrial genome (mtDNA).

**Figure 1:**
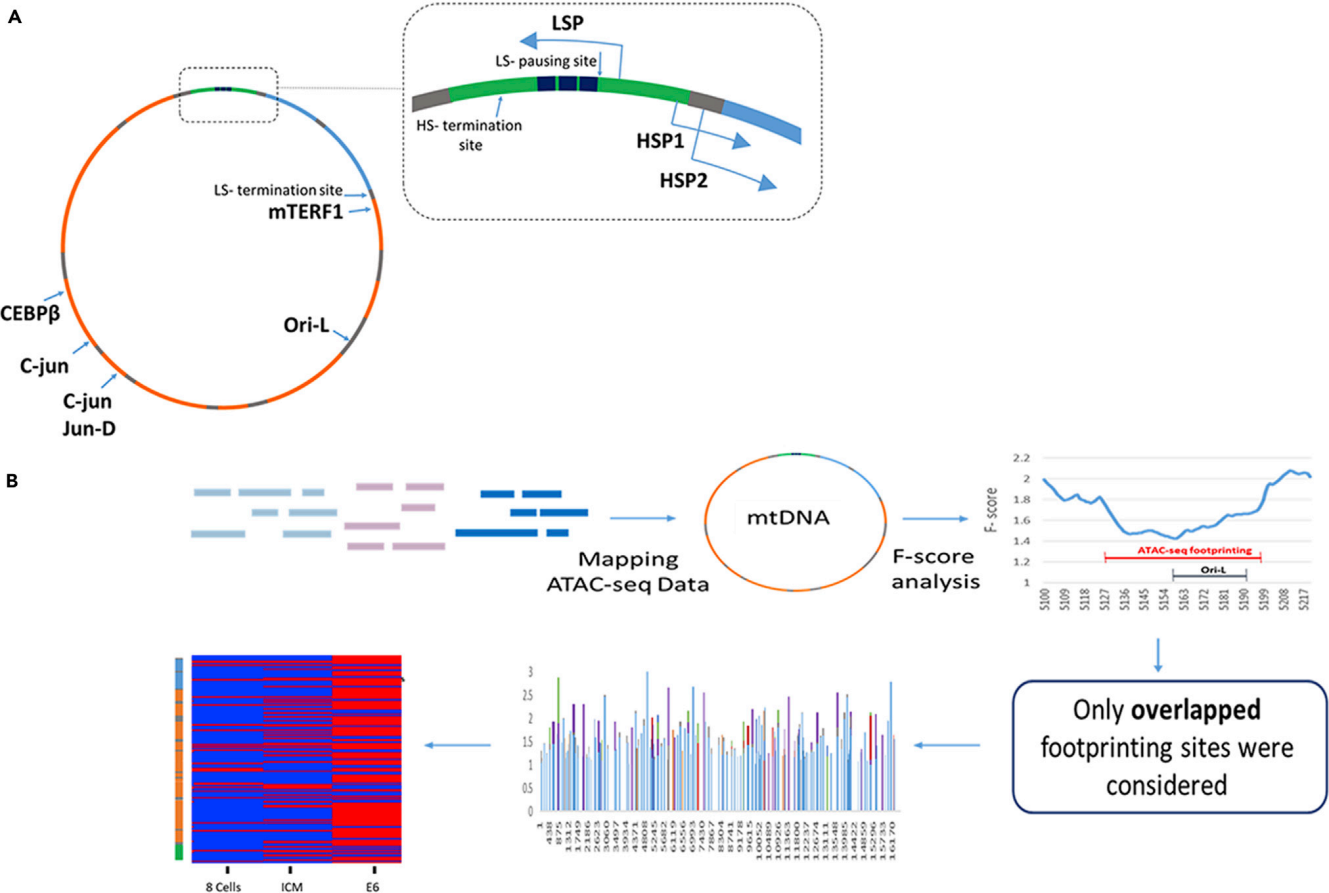
Workflow of mtDNA ATAC-Seq Data Analysis (A) Human mtDNA map. Light blue panels represent rRNA. Orange panels represent protein-coding genes. Gray panels represent tRNA genes. Green panel represent the D loop. Blue boxes represent the three conserved sequence blocks (CSBs). The dashed box highlights the D loop and known mtDNA promotors. (B) Workflow describing the identification of mtDNA ATAC-seq footprinting (ASFP) sites. Available ATAC-seq data from mouse embryos were downloaded from the Gene Expression Omnibus (GEO) database (see Transparent Methods). ATAC-seq reads were mapped onto the mouse mtDNA sequence, and read coverage per base was calculated.

Unlike the nDNA, the mtDNA is present in multiple cellular copies that may differ in number (Akiyama and Okada, 1992) and sequence (Avital et al., 2012) between individuals and tissues. Mammalian mtDNA is mainly composed of coding sequences (∼93% in humans and mice), including 13 genes for protein subunits of four of the five OXPHOS protein complexes (i.e., OXPHOS complexes I, III–V), two rRNA genes (12S and 16S), and 22 tRNA genes. The non-coding mtDNA regions (the D loop and the light-strand origin of replication [OriL]) harbor most known regulatory elements of mtDNA transcription and replication (Figure 1). During the early 1970s, strand-specific mtDNA polycistron transcripts have been identified in human cells (Aloni and Attardi, 1971), which subsequently were also determined in the mouse mtDNA (Barshad et al., 2018, Battey and Clayton, 1978, Blumberg et al., 2017). This bacterial-like pattern of transcription urged many to isolate the core elements of mtDNA transcriptional regulation, namely, mitochondrial transcription factor A (TFAM) (Fisher et al., 1987, Parisi and Clayton, 1991), the mitochondrial RNA polymerase (POLRMT) (Reid and Parsons, 1971, Ringel et al., 2011), mitochondrial transcription factor B2 (Falkenberg et al., 2002, Gustafsson et al., 2016, Morozov et al., 2014), and the transcription termination factor, mTERF (Daga et al., 1993). It was suggested that one of the key factors in mtDNA transcriptional regulation, TFAM, regulates mtDNA transcription and replication at low cellular concentrations, yet in higher concentrations it serves as the main (and possibly sole) mtDNA-coating protein (Ekstrand et al., 2004, Takamatsu et al., 2002), thus forming the bacterial-like DNA-protein structure, the nucleoid (Iborra et al., 2004, Kukat et al., 2015, Legros et al., 2004). Although it has been suggested that TFAM lacks binding sequence specificity, some researchers proposed mtDNA binding site preferences (Uchida et al., 2017), especially at regions that tend to adopt G-quadruplex structures (GQP) *in vitro* (Lyonnais et al., 2017) but not *in vivo* (Blumberg et al., 2018). Apart from TFAM, accumulating evidence reveals that known modulators of chromatin structure, such as MOF (Chatterjee et al., 2016), STAT3 (Macias et al., 2014), and SIRT1 (Aquilano et al., 2010), as well as other known regulators of nuclear gene transcription (Blumberg et al., 2014, She et al., 2011) (such as c-Jun, JunD, CEBPB, and MEF2D) are also imported into the mitochondria, bind the mtDNA, and regulate transcription. This raises the possibility that mtDNA higher-order organization is more structured and hence more regulated than once thought. Thus, we hypothesized that during the past ∼2 billion years of endosymbiosis, the mitochondria has retained some bacteria-like characteristics of genome packaging, yet additionally has adapted (at least in part) to the host higher-order DNA-protein organization (Barshad et al., 2018). By analyzing DNase-seq experiments from 324 human cell type samples, we recently found a conserved DNase genomic footprinting (DGF) pattern, with 29 mtDNA DGF (mt-DGF) sites common to >90% of the samples analyzed (Blumberg et al., 2018). Our analysis of publicly available TFAM chromatin immunoprecipitation sequencing experiments (ChIP-seq) in HeLa cells (Wang et al., 2013) suggest that these mt-DGFs are poor in TFAM binding, suggesting a more complex and regulated higher-order organization of the mtDNA (Blumberg et al., 2018). Taken together, these findings led us to hypothesize that the human mtDNA is subjected to higher-order protein-DNA organization, which is likely under tight regulation and is physiologically important. Nevertheless, it is unclear when during development such mtDNA organization is formed, and whether it is dynamic and associated with mitochondrial activity.

Recent analysis of experiments generated using assay for transposase-accessible chromatin with high-throughput sequencing (ATAC-seq) revealed footprinting site pattern, reflecting the dynamics of genome-wide chromatin accessibility during mouse preimplantation embryo development (Wu et al., 2016). This study encouraged us to extend such approach to investigate the accessibility pattern within the mtDNA during mammalian development.

Here, by analyzing ATAC-seq experimental data we found a dynamic mtDNA footprinting pattern during mouse and human embryogenesis. Specifically, we noticed progressive accumulation in the density of mtDNA footprinting sites during the course of mouse and human embryogenesis. Notably, whereas some ATAC-seq footprinting sites (ASFPs) occurred in multiple stages, including those that overlapped known regulatory elements, other sites where stage specific. Taken together our study reveals for the first time a dynamic chromatin-like mtDNA organization during the course of early mammalian embryogenesis, which associates with regulatory sites of mtDNA transcription and replication.

## Results

### Identifying mtDNA ATAC-Seq Footprinting Patterns during Mouse Embryogenesis

**Figure 2:**
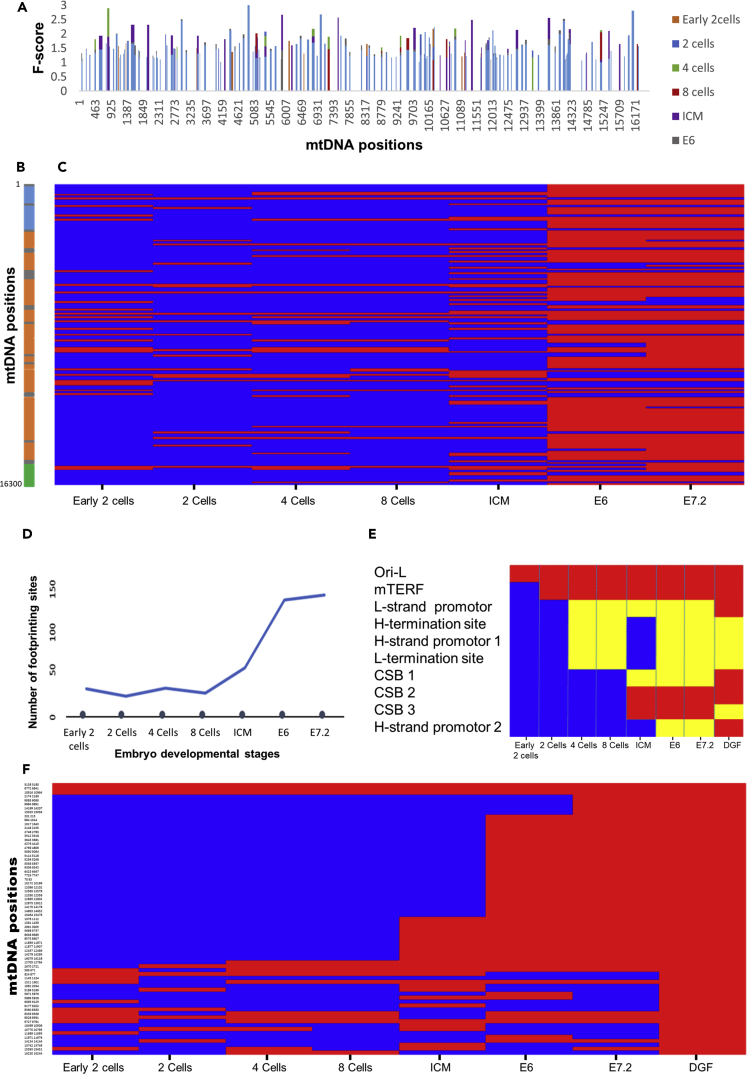
mt-ASFP Site Dynamics during Mouse Embryogenesis (A) A summary of overall mt-ASFP site distribution across the mouse mtDNA. Bars indicate mt-ASFP sites identified during each of the indicated developmental stages (see color code to the left of the panel). x axis, mtDNA positions. (B) Linear map of the mouse mtDNA (*Mus musculus*). Blue boxes represent rRNA genes. Orange boxes represent protein-coding genes. Gray boxes represent tRNA genes. Green panel represents the D loop. (C) Gradual increase in mtDNA-ASFP site distribution during the indicated embryonic developmental stage. Red, occupied sites; blue, unoccupied sites relative to previously analyzed stages. Notice that sites that remained unoccupied during all developmental stages (see A) were excluded. y axis, relative mtDNA position; x axis, developmental stage. (D) A graph demonstrating mt-ASFP site dynamics during mouse embryogenesis. (E) Heatmap demonstrating mt-ASFP site association with known mtDNA regulatory elements during mouse embryogenesis. Red, positive association; blue, negative association; yellow, an mt-ASFP site was identified < 40 bp from the indicated mtDNA regulatory site. (F) Heatmap representing all mt-ASFP sites that co-localize with mt-DGF sites identified in at least 4 out of 43 (>10%) analyzed mouse cell lines. y axis, mtDNA positions; x axis, developmental stage.

As a first step toward characterizing mtDNA protein-DNA organization during embryogenesis, we analyzed publicly available experimental ATAC-seq data from mouse embryos (Wu et al., 2016). To identify ASFPs in the mtDNA (mt-ASFPs), we slightly modified our previously described approach (Blumberg et al., 2018), also used for earlier analysis of mtDNA footprinting sites (Mercer et al., 2011). In brief, we calculated an F-score for each mtDNA position in sliding windows of variable sizes with a maximum of 124 bases and a minimum of 18 bases (see Transparent Methods for details, and Figure 1). To control for possible Tn5 digestion bias we used a recently published tool (Martins et al., 2018) (also see Transparent Methods and Table S1). In brief, we screened for possible sequence bias in ATAC-seq reads, in a six-nucleotide window size (K-mer), and did not identify any digestion bias. To reduce noise and ensure that reproducible data were being used, we restricted our analysis to those samples for which experimental duplicates were reported. Each of the duplicates was separately analyzed, and only mt-ASFPs shared by the duplicates were considered in subsequent analyses. To increase the stringency of our comparison of mt-ASFP dynamics during mouse development, sites that overlapped at least in one nucleotide position were merged. Such a stringent approach was applied to avoid false identification of mt-ASFP site changes and dynamics, thus reducing potential noise (see Transparent Methods for details). This approach was applied to ATAC-seq experimental data collected from mouse pre-implanted embryos at the following developmental stages: early 2-cell, 2-cell, 4-cell, 8-cell, and inner cell mass (ICM) stages (Wu et al., 2016), as well as from mouse embryonic day 6 (E6) and E7.2 embryos from another dataset (Neijts et al., 2016). Our results revealed increased density of the mt-ASFP site pattern during the course of embryogenesis (Figures 2 and S1, Table S2). The total number of mt-ASFP sites in all the tested mouse embryonic stages was 156, with 28 sites identified in early 2 cells, 21 sites during 2 cells, 24 sites in 4 cells, 29 sites in 8 cells, 49 sites in ICM, 117 sites in E6, and 122 sites in E7.2. Interestingly, in pre-implanted embryos (early 2-cell stage, ICM), the mt-ASFP pattern was relatively scarce, with both gain and loss of sites. Dramatic increases in the density of mt-ASFP sites were observed in both post-implanted stages (E6, E7.2), which also shared many such sites among each other, i.e., many sites were deemed common to both stages (Figures 2 and S1). Closer inspection revealed several types of mt-ASFP sites: first, a core set of mt-ASFP sites was shared by all embryonic stages (3% of mt-ASFP). Second, we identified a group of mt-ASFP sites that appeared only after several cell divisions, yet persisted in all subsequent developmental stages (64% of mt-ASFP). Third, we noticed mt-ASFP sites that alternately appeared during the course of embryogenesis (13% of mt-ASFP), and finally, there was a group of mt-ASFP sites that appeared only during certain pre-implantation stages (20% of mt-ASFP) (Figure S1A). Notably, although the ATAC-seq experiments of pre- and postimplantation mouse embryos were generated by two different laboratories, they used virtually the same experimental protocol. Second, the persistence of certain mt-ASFP sites during all tested stages attests for the negligible impact of CRISPR-based mtDNA depletion on the analysis. Nevertheless, we cannot exclude some fluctuations that could be resolved only upon analysis of ATAC-seq data generated by a single source.

In addition to changes in mt-ASFP site density, we also observed that the distribution of the mt-ASFP sites throughout the mtDNA varied across the embryonic stages. Although there were no significant differences in the distribution of sites within the protein-coding rRNA and tRNA genes during pre-implantation, the presence of mt-ASFP sites in the D loop was detected only during the 4-cell and 8-cell stages, as well as during post-implantation stages E6 and E7.2 (Figure S1B). This implies that certain regulatory elements become occupied during development after zygote formation. Next, we analyzed mt-ASFP site gain or loss in each of the embryonic mouse stages. Although there was a gradual increase in unique non-redundant sites during mouse development, there was no unique pattern of mt-ASFP site loss (Figure S1C). These results are consistent with the progressive establishment of a distinct mt-ASFP site pattern relatively early during mouse embryogenesis. Notably, it would be of interest to assess mt-ASFP site pattern in the oocyte, which will shed light on the dynamics of the pattern of mtDNA footprinting pre-fertilization.

Recently, we showed that the identification of footprinting sites is comparable between ATAC-seq and DNase-seq experiments (Blumberg et al., 2018). Hence, to validate the results of ATAC-seq data analysis by an alternative approach, we analyzed available DNase-seq data from early mouse embryos (2-cell, 4-cell, and 8-cell stages and the morula) (Lu et al., 2016). Such analysis revealed that all mt-ASFP sites that overlapped with known regulatory elements were also identified as mt-DGF (Table S3 and Table S4, Figure S1D). Notably, similar to the ATAC-seq analysis, a gradual increase in the number of mt-DGF sites was observed during the course of embryogenesis (Figure S1D), thus supporting the robustness of our observation and further attesting to the negligible impact of CRISPR-based mtDNA depletion on our analysis (see also Figure S2 for ATAC-seq read coverage). Finally, we excluded possible digestion bias in both mt-ASFP and mt-DGF data using a recently developed tool (Martins et al., 2018, and see Transparent Methods). Thus, the observed progressive mtDNA occupation during the course of embryogenesis is robust, regardless of the analysis method used.

### Nuclear Mitochondrial DNA Fragments Are Not Enriched in Mouse mt-ASFP Sites

It is plausible that the mtDNA ATAC-seq reads used in the current study are contaminated by mtDNA fragments that were transferred into the nucleus during the course of evolution, known as nuclear mitochondrial pseudogenes (nuclear mitochondrial DNA fragments [NUMTs]) (Hazkani-Covo et al., 2003, Mishmar et al., 2004). Notably, as mt-ASFP sites are defined by reduced number of reads at a given site, these might be affected by excess of reads that were mapped to both the nucleus and to active mtDNA. We, therefore, conducted a comprehensive screen to assess the number of NUMT-associated reads per sample per mtDNA position in the ATAC-seq data. To facilitate such a screen, we used a previously published collection of NUMT variants recorded over the entire mouse mtDNA (Calabrese et al., 2012). In general, the proportion of reads harboring NUMT variants in mouse comprised an average of only 0.195% of the reads (SD = 0.48%). Furthermore, the proportion of NUMT reads was not statistically different between mt-ASFP sites and non-ASFP sites across the entire human mtDNA (ASFP sites = 0.18%, SD = 1.57%; non- ASFP sites = 0.263%, SD = 0.54%; Figure S1E). We thus conclude that NUMT reads had only a negligible impact on our ATAC-seq analysis.

### Common ATAC-Seq Footprinting Sites Co-localize in Part with Known mtDNA Regulatory Elements

As an initial step toward assessing the functional importance of mt-ASFP sites during mouse embryogenesis, we screened for association between the mt-ASFP sites and mtDNA elements of regulatory importance (Blumberg et al., 2017, Chang and Clayton, 1985) (Figure 2A). First, we noted that the above-mentioned core set of mt-ASFP sites, detected at all embryonic stages, includes the light-strand OriL (Figure 2E). Second, we found that the group of mt-ASFP sites that appeared and persisted after several cell divisions included most known regulatory elements, other than the light-strand promoter (Figure 2). Specifically, the three conserved sequence blocks (CSBs) overlapped mt-ASFP sites that appeared during the ICM stage and persisted in subsequently analyzed stages. In addition, the binding sites for mTERF associated with an mt-ASFP site that appeared as early as during the 2-cell stage, and persisted in all subsequent stages. The mt-ASFP sites that overlapped H-strand promoter 2 (HSP2) appeared only during post-implantation (i.e., the E6 and E7.2 stages). Finally, we identified a group of mt-ASFP sites that displayed alternate appearance pattern. These included those sites that overlapped the HSP1, light strand (L strand ) transcription termination site, and heavy strand (H strand) transcription termination site, which appeared during the 4- to 8-cell stages, were not apparent during the ICM, but re-appeared during post-implantation (Figure 2E). Taken together, the findings show that whereas certain sites that overlap with regulatory sites became occupied even during pre-implantation, others became occupied only later (Figures 2E and 2F).

### Secondary Structures Coincide with Common ATAC-Seq Footprinting Sites

It has been suggested that guanine-rich sequences in human mtDNA tend to form GQPs, which potentially affect mtDNA replication initiation and genome stability (Chen et al., 2008, Dong et al., 2014). Recently, by analyzing DNase-seq experiments in 324 human cell types, we found that >90% of the samples shared 29 mt-DGFs, which associated with GQP-forming sequences (Blumberg et al., 2018). This finding encouraged us to assess the association of GQP-forming sequences with the identified mouse mt-ASFP sites. We found that of the 81 sequences with the propensity to adopt GQPs, 25 co-localized with mt-ASFP sites, a value statistically different from random (p < 0.01, chi-square test). To further characterize the association of mt-ASFP sites with sequences that potentially form secondary structures, we also considered the association of mt-ASFP sites with tRNA sequences. Such analysis revealed that 21 of the 22 mtDNA-encoded tRNA genes co-localized with mt-ASFP sites, a value that also differed from what would be expected by chance (p < 0.001, chi-square test). As secondary structures associate with a variety of regulatory features, the significant co-localization of a subset of mt-ASFP sites with secondary structures we observed here further supports the functional importance of such sites.

### Mouse Embryonic mt-ASFP Sites Are Also Occupied in Adult Cells

As mtDNA becomes increasingly coated during the course of mouse embryogenesis, we asked whether any of these apparently occupied sites in the mtDNA persist until adulthood. To address this question, we initially examined publicly available DNase-seq experimental results from 43 different mouse adult cell lines (ENCODE consortium) (see Transparent Methods). This revealed a total of 178 mt-DGF sites, of which more than 65% (N = 122) were shared by at least 10% of the cell lines (Table S5; Blumberg et al., 2018). We thus conclude that whereas certain mt-DGF sites are common to many cell types, others are cell line specific. A comparison of the precise mtDNA positions of these mt-DGF sites (in adult tissues) to the overall set of mt-ASFP sites identified during embryogenesis revealed that 69 of 122 “adult” mt-DGF sites (found in at least 10% of the analyzed adult samples) overlapped with “embryonic” mt-ASFP sites. Notably, 82% of these sites were identified during the post-implantation E6 and E7.2 stages (Figure 2, Table S2), suggesting that at least some of the mtDNA sites that were occupied during embryogenesis remained so until adulthood. Strikingly, the mt-DGF sites in adult cells that overlapped with mt-ASFP sites identified during embryogenesis harbored all known regulatory elements (Figures 2E and 2F). These findings suggest that establishment of the adult mt-ASFP sites pattern begins during differentiation.

### mtDNA ATAC-Seq Footprinting Dynamics during Human Embryogenesis

**Figure 3:**
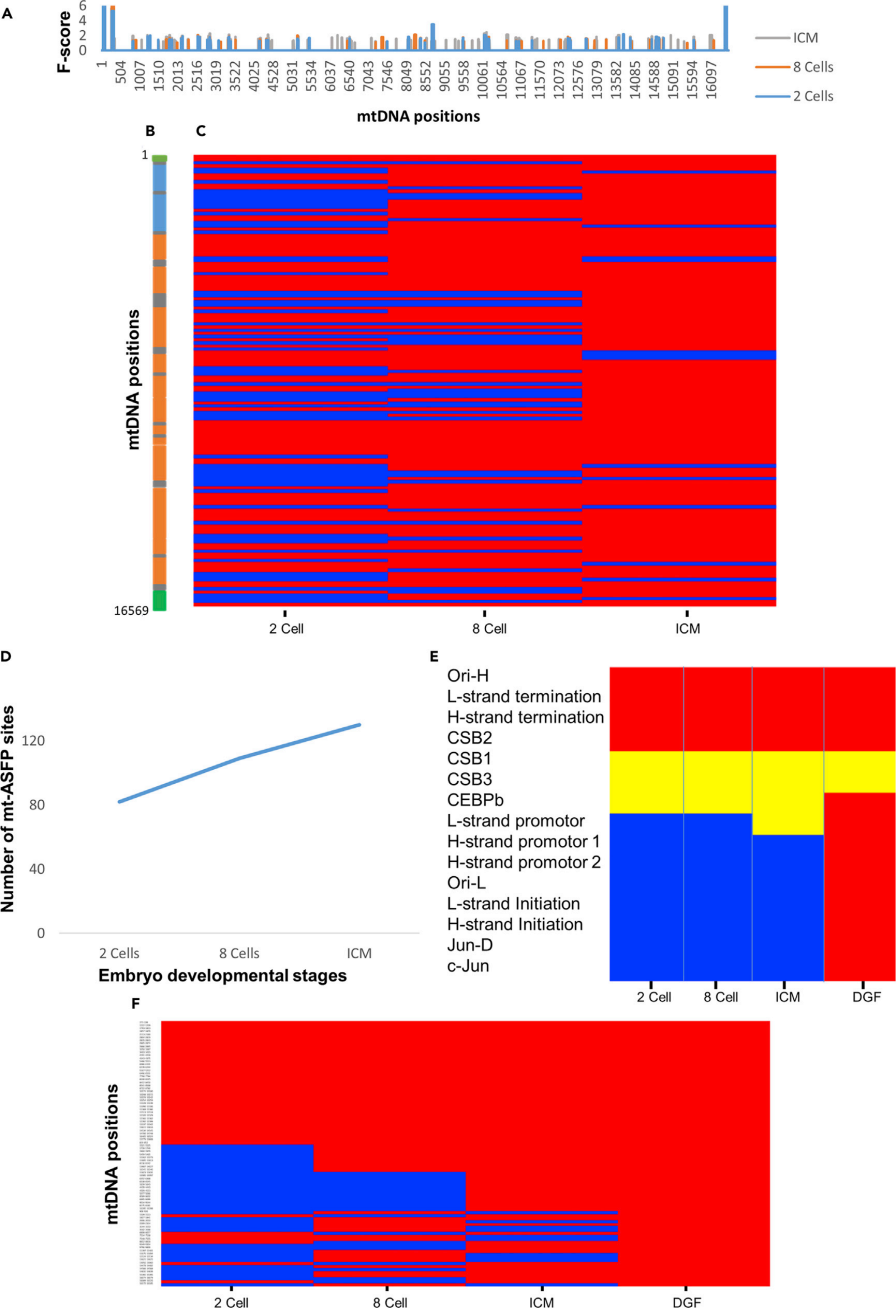
: mt-ASFP Site Dynamics during Human Embryogenesis (A) A summary of overall mt-ASFP site distribution across the human mtDNA. Bars indicate mt-ASFP sites identified during each of the indicated developmental stages (see color code to the left of the panel). x axis, mtDNA positions. (B) Linear map of the human mtDNA. Blue boxes represent rRNA genes. Orange boxes represent protein-coding genes. Gray boxes represent tRNA genes. Green panel represents the D loop. (C) Gradual increase in mt-ASFP site distribution during the indicated human embryonic developmental stages. Red, occupied sites; blue, unoccupied sites relative to previously analyzed stages. Notice that sites that remained unoccupied during all developmental stages (see A) were excluded. y axis, relative mtDNA position; x axis, developmental stage. (D) A graph demonstrating mt-ASFP site dynamics during human embryogenesis. (E) Heatmap representing the association of mt-ASFP sites with known mtDNA regulatory elements during human embryogenesis. Red, positive association; blue, negative association; yellow, an mt-ASFP site was identified <40 bp from the indicated mtDNA regulatory site. (F) Heatmap representing all mt-ASFP sites associated with mt-DGF sites in 70 adult human samples (>10% of the samples).

We next asked whether the dynamic mt-ASFP sites pattern seen during mouse embryogenesis also exists in other mammals. To address this question, we analyzed the results of recently published ATAC-seq experiments in human pre-implantation embryos (Wu et al., 2018). ATAC-seq experimental data were collected from human 2-cell- and 8-cell-stage embryos, as well as from the ICM stage, and the analysis was performed using the same protocol as used for mouse embryos mentioned above (see Transparent Methods). The total amount of mt-ASFP sites during all the tested human embryogenesis stages was 143, with 82 sites identified in 2 cells, 109 sites in 8 cells, and 130 sites in ICM. Therefore, our results revealed similar dynamics of the human mt-ASFP sites pattern as observed during mouse mtDNA embryogenesis, namely, increased density of the mt-ASFP landscape (i.e., including both overlapping sites among stages and non-redundant sites) over the course of human embryogenesis (Figures 3 and S3 and Table S6). Second, human mtDNA displayed similar types of mt-ASFP sites as seen with mouse mtDNA (Figure 3C): a core set of mt-ASFP sites that was shared by all embryonic stages (52% of mt-ASFP), a group of mt-ASFP sites that appeared only after several cell divisions, yet persisted in all subsequent developmental stages (10% of mt-ASFP), and mt-ASFP sites that alternately appeared during the course of embryogenesis (38% of mt-ASFP). Third, as in mouse, we utilized a comprehensive list of human NUMTs (Li et al., 2012) and found that the proportion of NUMT reads was not statistically different between mt-ASFP sites and non-ASFP sites across the entire human mtDNA (ASFP sites = 0.16%, SD = 1.32%; non- ASFP sites = 0.195%, SD = 0.46%; Figure S1F). Thus, human NUMT reads had only little impact on our ATAC-seq analysis. Despite the similarities in the dynamics of mt-ASFP sites between mice and humans, it is noteworthy that whereas human mtDNA was already highly occupied during the pre-implantation stages addressed, a similar degree of mt-ASFP site density was observed only during post-implantation stages in mouse (Figures 2D and 3D). Taken together, mouse and human mt-ASFP patterns showed very similar dynamics, thus suggesting that the establishment of higher-order mtDNA organization during embryogenesis is conserved in mammals.

Next, we asked whether some of the human mt-ASFP sites persisted from the embryonic stage to adulthood. Accordingly, publicly available DNase-seq experimental results from 70 different human cell samples (ENCODE consortium) were consulted (see Transparent Methods) (Blumberg et al., 2018). This analysis revealed a total of 144 mt-DGF sites, which were shared by at least 10% of the samples (Table S7) (Blumberg et al., 2018). While comparing the precise mtDNA positions of mt-DGF sites (in samples from adult tissues) with the overall set of mt-ASFP sites identified during embryogenesis, we found that 88 of 145 adult mt-DGF sites overlapped with “embryonic” mt-ASFP sites. Notably, 88% of these sites could be identified as early as during pre-implantation (Figure 3F and Table S6), suggesting that at least some of the mtDNA sites that were occupied during embryogenesis persisted until adulthood. Similar to what was seen in mouse, our analysis revealed that the human mt-ASFP sites co-localized with known human mtDNA regulatory elements shared by all pre-implantation stages tested (Figure 3E). Among these sites were those overlapping with the heavy-strand origin of replication, heavy- and light-strand transcription termination sites, the three CSBs, and the CEBPβ-binding site. Furthermore, as in mouse, all the human embryonic mt-ASFP sites that were associated with known regulatory elements were such also in the adult (Figure 3E). Hence, in both humans and mice, the mt-ASFP pattern associates with known mtDNA regulatory elements, further supporting the potential regulatory importance of mt-ASFPs.

## Discussion

Our analyses of ATAC-seq and DNase-seq experiments from early embryonic stages in both mouse and human samples indicate gradual increase in the number of mtDNA footprinting sites during the course of mammalian embryogenesis. The consistency of the footprinting pattern between two different methods (ATAC-seq and DNase-seq), and between different organisms, attests for the robustness of this observation. Hence, higher-order mtDNA organization is likely more regulated and more dynamic than previously thought, even during embryogenesis. As such organization is already apparent during pre-implantation stages, and as the footprinting sites co-localize with known regulatory elements, it is tempting to suggest that regulatory activation of the mtDNA transpires during early embryogenesis.

The co-localization of our mt-ASFP sites with regulatory elements, particularly with sites that associate with mtDNA transcription, tempts to assess the connection between our observed footprinting dynamics during development and mtDNA transcription. With this in mind the recent discovery of TEAD4, a critical factor in mouse preimplantation embryogenesis, which also regulates mtDNA transcription in the trophectoderm, is noteworthy (Kumar et al., 2018). Once quantitative techniques that measure nascent RNA formation, such as PRO-seq (Kwak et al., 2013), which was recently adapted to mtDNA analysis (Blumberg et al., 2017), are adapted to single-cell analysis, one will be able to experimentally assess such correlation.

The similarity in mt-ASFP dynamics during mouse and human development, and partial similarity in distribution of mt-ASFP sites, raises the possibility that the two species share the underlying mechanism of mt-ASFP formation. This interpretation is further supported by the tendency of both mouse and human mt-ASFP sites to adopt secondary DNA structures (such as G-quadruplex) and co-localization with regulatory elements. It would be of great interest to assess whether certain regulatory factors tend to bind such sites *in vivo* in the mitochondria of both mouse and man. Nevertheless, as human mtDNA appears to be more occupied already during early embryogenesis than the mouse mtDNA, some mechanistic differences should be considered. Such interpretations should be studied in the future.

In summary, we have provided the first direct evidence for a dynamic chromatin-like organization of mtDNA during mammalian embryogenesis. Specifically, we found that both mouse and human mtDNA display ASFP sites that precisely overlap with known mtDNA regulatory elements. These footprinting sites emerge gradually during embryogenesis and exhibit similar dynamics in both species. Furthermore, in both mammals, we identified mt-ASFP sites that were occupied during all developmental stages, sites that emerged and are maintained in subsequent stages after their appearance, transient sites that emerged but are not retained, and finally, stage-specific sites. Such similarity in the dynamics of mt-ASFP sites, in conjunction with their co-localization with known regulatory sites, suggests the physiological importance of these mt-ASFP sites in mammalian development.

### Limitations of the Study

• Although pre-implantation embryonic stages were analyzed in both human and mice, post-implantation stages were analyzed only in mice. These should be analyzed only upon availability of such data.
• The analysis of ATAC-seq data was limited to human and mice embryogenesis; it would be interesting to analyze ATAC-seq data from carefully sampled embryonic stages in non-mammalian vertebrates and invertebrates.
• Some of the analyzed ATAC-seq data originated from Wu et al., who used CARM to deplete mtDNA reads. Despite such approach, we found that multiple mtDNA reads remained and that the gradual increase in mtDNA footprinting sites was observed not only in the ATAC-seq data, but in DNase-seq experiments from the same stages.
• Although ATAC-seq footprinting sites in mouse and human mtDNA associated with known transcriptional and replication regulatory elements, the functional impact of the majority of the identified footprinting sites is yet to be investigated.

## Methods

All methods can be found in the accompanying Transparent Methods supplemental file.

## Supplemental Information

Document S1. Transparent Methods, Figures S1–S3, Tables S1, S4, S6, and S7

Table S2. Calculated F-Scores for Mouse mt-ASFP Sites Per Mouse Embryonic Stage, Related to Figure 2 Last column represents mt-DGFs that co-localized with mt-ASFP sites.

Table S3. Calculated F-Scores for Mouse mt-DGF Sites Per Embryonic Stage, Related to Figure 2

Table S5. mt-DGF Sites in Mouse Adult Cell Lines, Related to Figure 2 mt-DGF sites that were present in more than 10% of the analyzed cell lines (N = 43) (according to Blumberg et al., 2018; Genome Research).
