# Supplementary material for: mtDNA Chromatin-like Organization Is Gradually Established during Mammalian Embryogenesis
Source: iScience. 2019 Jan 8;12:141–51. doi: 10.1016/j.isci.2018.12.032 (PMC6352746; doi:10.1016/j.isci.2018.12.032)
Supplement: Document S1. Transparent Methods, Figures S1–S3, Tables S1, S4, S6, and S7 [file mmc1.pdf]

ISCI, Volume *12*

Supplemental Information

mtDNA Chromatin-like Organization Is Gradually Established during Mammalian Embryogenesis

Shani Marom, Amit Blumberg, Anshul Kundaje, and Dan Mishmar

Marom et al 2018: Inventory of Supplemental Information

Transparent Methods: Experimental Procedures.

Supplemental Figures

Supplemental Figure S1, related to figures 2-3: Dynamics of mt-ASFP sites and their distribution during mouse embryogenesis. A. Pie chart summarizing the distribution of mt-ASFP site prevalence during mouse embryonic stages. Blue area - ASFP sites shared by all embryonic stages tested (3% of ASFP). Light blue area - ASFP sites that appeared only after several cell divisions, yet persisted in all subsequent developmental stages (64% of ASFP). Gray area - ASFP sites that appeared only during certain pre-implantation stages (20% of ASFP). Orange area - ASFP sites with alternating appearance during embryogenesis (13% of ASFP). B. Graph illustrating the genomic distribution of the ASFP sites across the different mtDNA regions. C. Bar graph demonstrating ASFP site gain/loss, as compared to ASFP site distribution in the preceding embryonic stage. D. A graph demonstrating mt-DGF site dynamics during mouse embryogenesis. E. A diagram comparing the prevalence of NUMT reads at ASFP versus non-ASFP mtDNA sites. F.A diagram comparing the prevalence of human NUMT reads at ASFP versus non-ASFP mtDNA sites.

Supplemental Figure S2, related to figures 2-3: Mouse ASFP site length, read coverage and ATAC-seq site read coverage and F-score data. A. Box plot representing mt-ASFP site length distribution. Average length is indicated. B. A table summarizing the average, median, maximum and minimum mt-ASFP site lengths at each embryonic stage tested. C. Graph representing the read coverage for a representative mouse ATAC-seq experiment. Notice that the reduced read coverage in the end of the linearized mtDNA is presented prior to re-mapping to correct for the circularity of the molecule (applies also to Figure S3). X axis - mtDNA nucleotide positions, Y axis - number of reads per site. D. Distribution of calculated F-scores in a representative mouse sample. X axis - the mtDNA nucleotide positions, Y axis - F-scores per position. E. Graph representing specific F-score data for the Ori-L site (underlined in red).

Supplemental Figure S3, related to figures 2-3: Human mt-ASFP site length, read coverage and ATAC-seq site read coverage and F-score data. A. Box plot representing mt-ASFP site length distribution. Average length is indicated. B. A table summarizing the average, median, maximum and minimum of ASFP site lengths at each embryonic stage tested. C. Graph representing the read coverage for a representative human ATAC-seq experiment. D. Distribution of calculated F-scores in a representative human sample. X axis - the mtDNA nucleotide positions, Y axis - F-scores per position. E. Graph representing specific F-score data for the Ori-L site (underlined in red – similar to Figure S2).

Supplemental Tables

Supplemental Table S1, related to Figure 1 and Figure 3: Calculated chi-square for K-mer analysis. A. Table of chi-square analysis for mouse embryogenic stages. B. Table of chi-square analysis for human embryonic stages.

Supplemental Table S2, related to Figure 2: Calculated F-scores for mouse mt-ASFP sites per mouse embryonic stage. Last column represents mt-DGFs that co-localized with mt-ASFP sites.

Supplemental Table S3, related to Figure 2: Calculated F-scores for mouse mt-DGF sites per embryonic stage.

Supplemental Table S4, related to Figure 2: Mouse mt-DGF sites analysis as a validation for the results of mouse ATAC-seq experiments. mt-DGF co-localize with mt-ASFP sites at mouse mtDNA regulatory elements during early mouse embryogenesis. Plus/minus signs indicate whether our identified mt-ASFP or mt-DGF sites co-localize with known mtDNA regulatory elements. Asterisk - an mt-ASFP site located no more than 40 bp from the indicated regulatory elements.

Supplemental Table S5, related to Figure 2: mt-DGF sites in mouse adult cell lines. mt-DGF sites that were present in more than 10% of the analyzed cell lines (N=43) (according to Blumberg et al. 2018, **Genome Research**).

Supplemental Table S6, related to Figure 3: Calculated F-scores for mt-ASFP sites per mouse embryonic stage. The last column represents adult mt-DGFs that co-localize with mt-ASFP sites.

Supplemental Table S7, related to Figure 3: mt-DGF sites in human adult cell lines. mt-DGF sites that were present in more than 10% of the analyzed cell lines (N=70) (according to Blumberg et al. 2018, **Genome Research**).

Experimental Procedures

Samples used for data analysis: ATAC-seq data for mouse embryogenesis were obtained from GEO Accession number GSE66582 (Neijts et al., 2016; Wu et al., 2016). DNase-seq data for mouse embryogenesis were obtained from GEO Accession number GSE76642 (Lu et al., 2016). While considering mouse preimplantation and post-implantation stages, we analyzed a total of 28 experiments, with each developmental stage represented by two biological replicates each performed in duplicates (i.e. 4 experiments per developmental stage). ATAC-seq data for human embryogenesis were obtained from GEO Accession number GSE101571 (Wu et al., 2018). While considering human samples, we analyzed a total of 12 experiments, with each developmental stage represented by two biological replicates (two independent samples), each performed in duplicates (i.e. 4 experiments per developmental stage). Notably, both ATAC-seq and DNase-seq data were obtained from whole cells, not isolated nuclei.

Sample-specific mtDNA sequence reconstruction and mapping, read coverage calculation and circular-like mapping of sample-specific mtDNA sequences: Analyses were performed as described previously (Blumberg et al., 2017). In brief, after mapping reads against the mtDNA reference genome (NCBI accession numbers NC_005089.1 (mouse) and NC_012920.1 (human)), sample-specific mtDNA reference genomes were re-constructed and ATAC-seq and DNase-seq reads were aligned while taking into account the circular organization of the mtDNA, as recently performed (Blumberg et al., 2017). Read coverage for each position was calculated using the 'genomecov' command in BEDtools (http://bedtools.readthedocs.org/en/latest/ version

2.25)(Quinlan and Hall, 2010) (for mouse, Figure S2; for human, Figure S3). Only positions covered by at least 300X read depth were taken into consideration. For ATAC-seq analysis, average, median, maximum and minimum coverage are presented (for mouse, Figure S2; for human, Figure S3).

ATAC-seq analysis: Publically available Sequence Reads Archive (SRA) files were converted into a FASTq format using sratoolkit (www.ncbi.nlm.nih.gov/Traces/sra/?view=toolkit_doc). The sequencing adaptors were trimmed by applying an ATAC-seq analysis pipeline, specifically using 'function detect adaptors' and 'function trim adaptors' (http://github.com/kundajelab/atac_dnase_pipelines). mt-ASFP sites were identified using a similar set of criteria as recently described for the identification of DNase footprinting sites (Blumberg et al., 2017). In brief, for each mtDNA nucleotide position, an F-score was calculated in sliding read windows of ~120 bp using the following equation: $F = (C + 1)/L + (C + 1)/R$, where C represents the average number of reads in the central fragment, L represents the average read count in the proximal fragment, and R represents the average read count in the distal fragment. The lowest F-scores were interpreted as reflecting ASFP sites. To identify overlapping mt-ASFP sites in experimental replicates, we employed the BEDtools command 'intersect' using the '-a' and '-b' options for each of the different replicates. Only mt-ASFP sites that were common to all replicates of a given experiment (i.e. overlapping by at least 1 nucleotide position) were used for subsequent analysis. In addition, in cases where triplicates were available, we first recorded overlapping sites in two of them using BEDtools, followed by assessment of overlapping sites with the third replicate. For analysis of mt-ASFP sites dynamics,

we considered mt-ASFP sites that overlap in all embryonic stages analyzed. To this end, mt-ASFP data from all developmental stages were combined in all analyzed samples. The length of each combined mt-ASFP site was measured between the 5' and 3' nucleotide positions of the most proximal and distal overlapping mt-ASFP sites, respectively.

Statistical comparison between BED files: Statistical comparison between sets of sites represented in BED format was performed using BEDtools (Quinlan and Hall 2010).

DNase-seq analysis: ENCODE DNase-seq fastq files were downloaded from the ENCODE consortium website: http://hgdownload.cse.ucsc.edu/goldenPath/mm9/encodeDCC/wgEncodeUwDnase/). mt-DGF sites were identified by following a method similar to the approach described in the above sub-section 'ATAC-seq analysis'(Blumberg et al., 2018).

K-Mer analysis: To control for possible Tn5 digestion bias we screened for possible sequence bias in ATAC-seq and DNase-seq reads, in 6 nucleotide window size (K-mer). K-mer scores were calculated using the seqOutBias pipeline (https://github.com/guertinlab/seqOutBias) as previously reported (Martins et al., 2018). In brief, seqOutBias was applied to BAM files available from all tested human and mouse ATAC-seq data files, and k-mer values were calculated for each mtDNA position per sample. Next, we used these k-mer values (per mtDNA position) to calculated average and standard deviation. Then, we tested whether the distribution of k-mer values in mt-ASFP sites recorded for all embryonic stages deviated from

the mean values (+SD) calculated for the entire mtDNA per sample; by that we referred only to the mt-ASFP sites that obtained across all the tested embryonic stages). In order to do so, we asked whether the K-mer calculated values in the mtDNA ASFP sites significantly deviated by at least two standard deviations from the mean K-mer values calculated for randomly selected mtDNA sites (chi square test). Such comparison was performed for each of the tested samples.

NUMT analysis: The proportion of NUMT reads was estimated using bam-readcount (https://github.com/genome/bam-readcount) by counting mtDNA-mapped ATAC-seq reads (within BAM files) that contained NUMT variants. NUMT genetic variants was obtained from the published collection of 150 mouse NUMT variants (Calabrese et al., 2012). For each analyzed sample, a sample-specific NUMT collection was generated by screening the reconstructed sample-specific mtDNA sequences. The proportion of sequencing reads harboring a specific NUMT mutation was estimated per mtDNA position.

Prediction of the potential of DNA sequences to adopt a G-quadruplex structure: G-quadruplex DNA structures were predicted for both the light and heavy mtDNA strands using QGRS Mapper (http://bioinformatics.ramapo.edu/QGRS/index.php)(Kikin et al., 2008). We used the previously published prediction parameters (Dong et al., 2014) (GQP max length = 33. Min; G-group size = 2; loop size = 0 to 36). mtDNA site coordinates were listed after merging the predicted site coordinates for both mtDNA strands. Statistical assessment of mt-ASFP site enrichment within sequences with the propensity to adopt secondary structure (GQP and mtDNA-encoded tRNAs, separately) was compared to their association with a set of random

non-ASFP mtDNA sites of comparable sequence length to the average mt-ASFP site (Chi square test for goodness of fit).

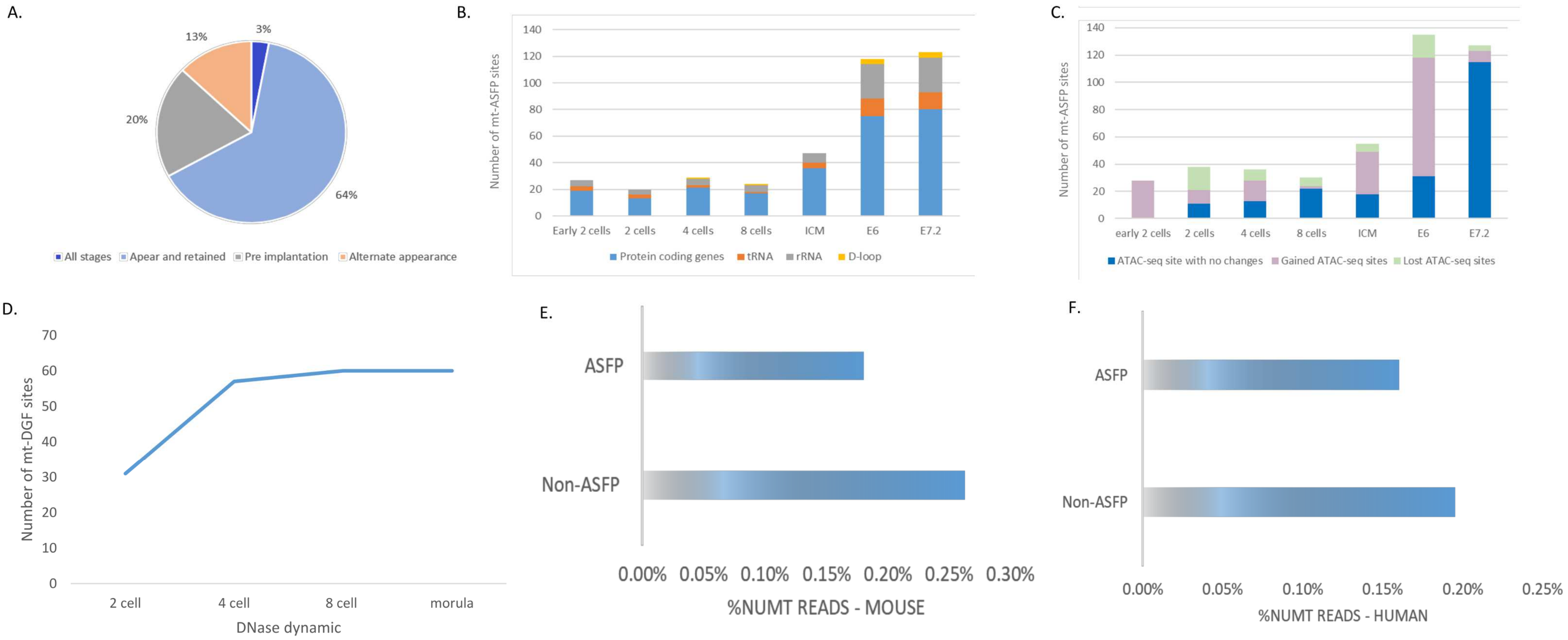

Supplemental Figure S1, related to Figures 2-3: Dynamics of mt-ASFP sites and their distribution during mouse embryogenesis. A. Pie chart summarizing the distribution of mt-ASFP site prevalence during mouse embryonic stages. Blue area - ASFP sites shared by all embryonic stages tested (3% of ASFP). Light blue area - ASFP sites that appeared only after several cell divisions, yet persisted in all subsequent developmental stages (64% of ASFP). Gray area - ASFP sites that appeared only during certain pre-implantation stages (20% of ASFP). Orange area - ASFP sites with alternating appearance during embryogenesis (13% of ASFP). B. Graph illustrating the genomic distribution of the ASFP sites across the different mtDNA regions. C. Bar graph demonstrating ASFP site gain/loss, as compared to ASFP site distribution in the preceding embryonic stage. D. A graph demonstrating mt-DGF site dynamics during mouse embryogenesis. E. A diagram comparing the prevalence of NUMT reads at ASFP versus non-ASFP mtDNA sites. F.A diagram comparing the prevalence of human NUMT reads at ASFP versus non-ASFP mtDNA sites..

A.

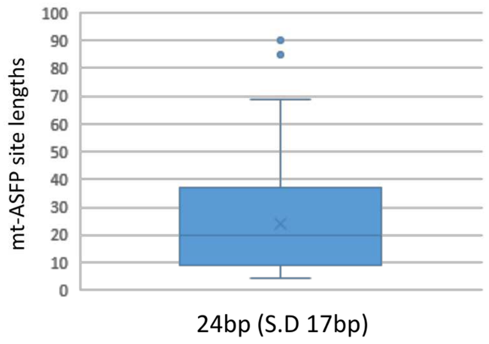

B.

	Early 2 cells	2 cells	4 cells	8 cells	ICM	E6	E7
**Average coverage**	1509348	725630	383893	78946	291629	62273	68869
**Median**	1237767	688781	386278	79224	293156	62508	69149
**Max coverage**	6156885	1588738	526590	110832	384044	87389	97552
**Min coverage**	8576	2923	336	471	1717	466	529

C.

D.

E.

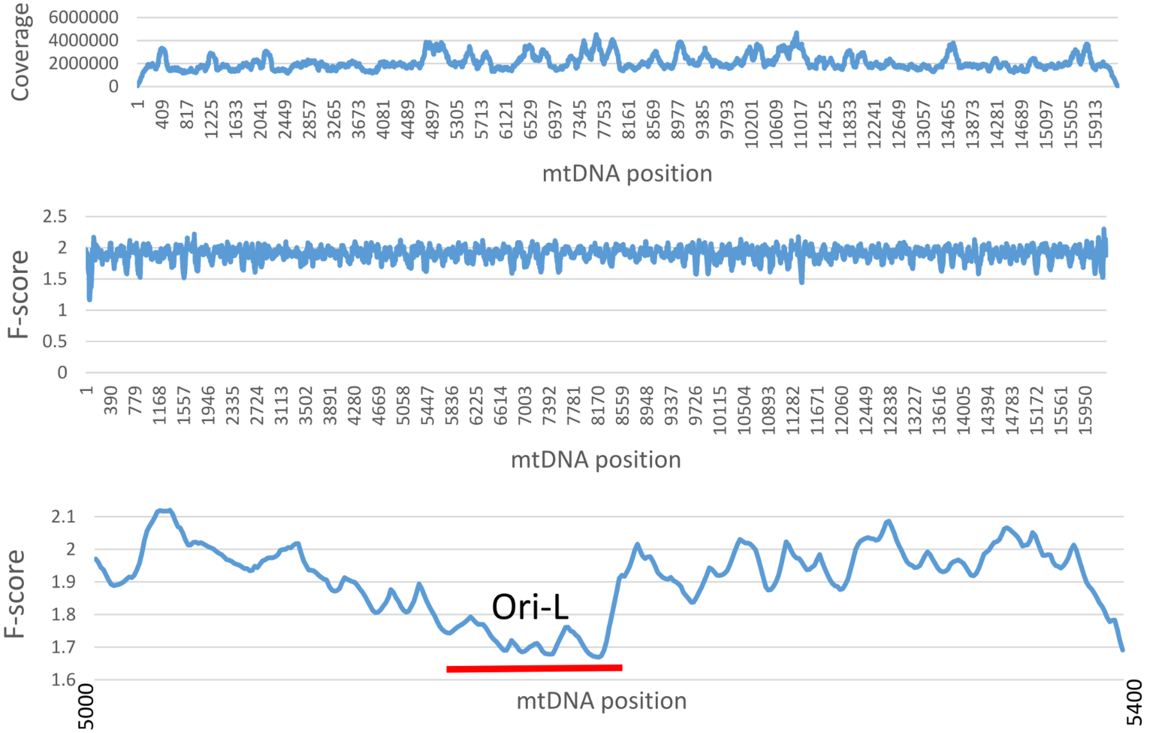

Supplemental Figure S2, related to Figures 2-3: Mouse ASFP site length, read coverage and ATAC-seq site read coverage and F-score data. A. Box plot representing mt-ASFP site length distribution. Average length is indicated. B. A table summarizing the average, median, maximum and minimum mt-ASFP site lengths at each embryonic stage tested. C. Graph representing the read coverage for a representative mouse ATAC-seq experiment. Notice that the reduced read coverage in the end of the linearized mtDNA is presented prior to re-mapping to correct for the circularity of the molecule (applies also to Figure S3). X axis - mtDNA nucleotide positions, Y axis - number of reads per site. D. Distribution of calculated F-scores in a representative mouse sample. X axis - the mtDNA nucleotide positions, Y axis - F-scores per position. E. Graph representing specific F-score data for the Ori-L site (underlined in red).

A

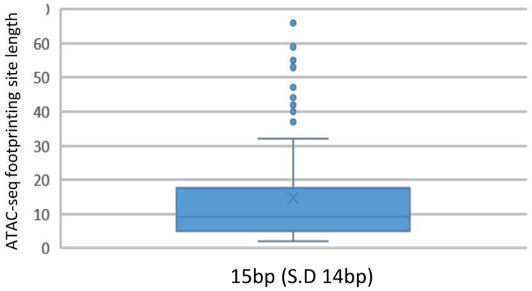

B.

	2 cells	8 cells	ICM
**Average coverage**	1393380	460614	332088
**Median**	1400438	466304	336495
**Max coverage**	1953866	659452	46683
**Min coverage**	1462	1104	811

C.

D.

E.

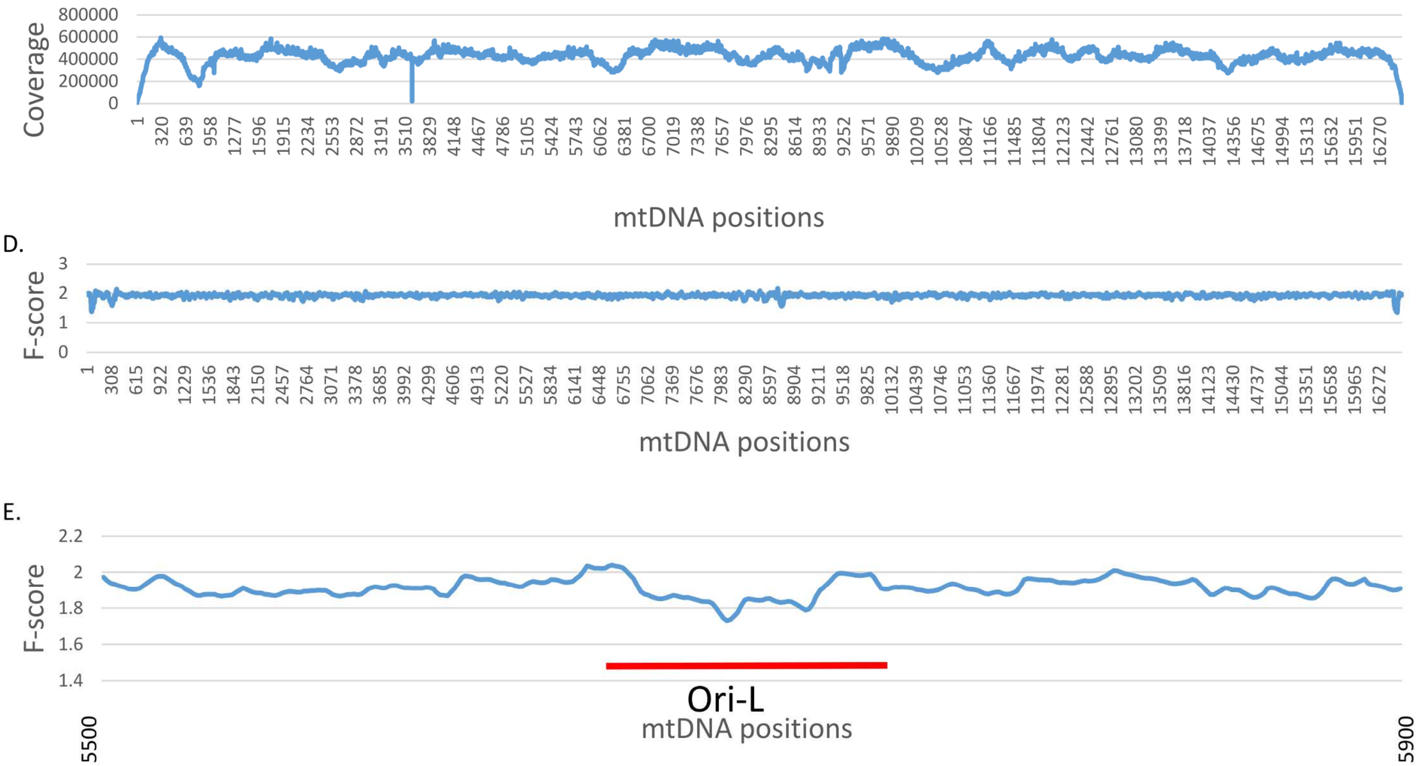

Supplemental Figure S3, **related to Figure 2-3: Human mt-ASFP site length, read coverage and ATAC-seq site read coverage and F-score data.** A. Box plot representing mt-ASFP site length distribution. Average length is indicated. B. A table summarizing the average, median, maximum and minimum of ASFP site lengths at each embryonic stage tested. C. Graph representing the read coverage for a representative human ATAC-seq experiment. D. Distribution of calculated F-scores in a representative human sample. X axis - the mtDNA nucleotide positions, Y axis - F-scores per position. E. Graph representing specific F-score data for the Ori-L site (underlined in red – similar to Figure S2).

A.	Mouse embryonic stage	Kmer within the ASFP sites (Observed)	Kmer within random mtDNA sites (maintain site sample size)	Total Kmer calculated
	Early 2 2 cells rep1	142	199	828
	Early 2 2 cells rep2	123	205	798
	2 cells rep 1	141	193	750
	2 cells rep 2	136	211	787
	4 cells rep 1	121	188	805
	4 cells rep 2	139	197	792
	8 cells rep 1	144	183	840
	8 cells rep 2	145	201	801
	ICM rep 1	132	198	838
	ICM rep 2	146	185	852
	E6 rep 1	135	189	720
	E6 rep 2	127	202	766
	E7.2 rep 1	119	196	785
	E7.2 rep 2	117	193	793

B.	Human embryonic stage	Kmer within the ASFP sites (Observed)	Kmer within random mtDNA sites (maintain site sample size)	Total Kmer calculated
	2 cells rep 1	72	735	1590
	2 cells rep 2	69	712	1483
	8 cells rep 1	62	698	1464
	8 cells rep 2	75	501	1435
	ICM rep 1	58	771	1500
	ICM rep 2	78	706	1401

Supplemental Table S1, related to Figure 1 and Figure 3: Calculated chi-square for K-mer analysis. A. Table of chi-square analysis for mouse embryogenic stages. B. Table of chi-square analysis for human embryonic stages.

Regulatory Element - DNAse	Position	2 cell	4 cell	8 cell	Morula
**mTERF1**	2660-2681	+	+	+	+
**L-strand origin of replication**	5160-5191	*	*	*	+
**CSB 1**	16035-16058	+	+	+	+
**CSB 2**	16089-16104	+	+	+	+
**CSB 3**	16114-16131	+	+	+	+
**L-strand promotor**	16183	*	*	*	*
**L-strand pausing site**	16159	*	*	*	*
**H-strand termination site**	16247	+	+	+	+
**H-strand promotor 2**	65	+	+	+	+
**H-strand promotor 1**	16282-16290	*	*	*	*
**L-strand termination site**	16284	*	*	*	*
**H-strand pausing site**	74	+	+	+	+

Regulatory Element - ASFP	Position	2 cells	4 cells	8 cells	ICM
**mTERF1**	2660-2681	+	+	+	+
**L-strand origin of replication**	5160-5191	+	+	+	+
**CSB 1**	16035-16058	-	-	-	*
**CSB 2**	16089-16104	-	-	-	+
**CSB 3**	16114-16131	-	-	-	+
**L-strand promotor**	16183	-	*	*	*
**L-strand pausing site**	16159	-	-	-	-
**H-strand termination site**	16247	-	*	*	-
**H-strand promotor 2**	65	-	-	-	-
**H-strand promotor 1**	16282-16290	-	*	*	-
**L-strand termination site**	16284	-	*	*	-
**H-strand pausing site**	74	-	-	-	-

Supplemental Table S4, related to Figure 2: Mouse mt-DGF sites analysis as a validation for the results of mouse ATAC-seq experiments. mt-DGF co-localize with mt-ASFP sites at mouse mtDNA regulatory elements during early mouse embryogenesis. Plus/minus signs indicate whether our identified mt-ASFP or mt-DGF sites co-localize with known mtDNA regulatory elements. Asterisk - an mt-ASFP site located no more than 40 bp from the indicated regulatory elements.

ATAC-seq sites		F-Score				ATAC-seq sites		F-Score			
Start	End	2 Cells	8 Cells	ICM	DGF>90%	Start	End	2 Cells	8 Cells	ICM	DGF>90%
51	105	5.64	5.38	1.13		9350	9355	1.12	0.00	1.50	
203	218	1.23	0.00	0.00		9372	9381	0.00	1.23	1.47	
274	345	3.40	6.10	5.68		9436	9442	1.11	1.03	1.25	
353	364	1.86	1.08	1.63		9535	9545	0.00	0.00	1.40	
820	852	1.35	1.34	1.93		9552	9584	1.37	1.22	1.74	
889	894	0.00	1.25	1.23		9626	9630	1.19	0.00	1.18	
909	927	0.00	1.41	0.00		9797	9801	0.00	0.00	1.32	
1069	1076	1.33	1.16	1.47		9963	9969	1.48	1.17	1.50	
1221	1230	1.69	1.82	2.07		9976	9991	1.03	0.00	1.14	
1268	1279	0.00	1.25	1.41		10050	10054	1.25	0.00	0.00	+
1294	1301	1.82	1.73	1.94		10071	10082	1.43	0.00	0.00	+
1361	1368	1.25	0.00	0.00		10112	10155	1.84	1.93	2.26	+
1510	1514	1.31	1.05	1.25		10175	10181	2.14	1.86	2.46	
1522	1526	0.00	1.03	1.28		10200	10216	1.31	1.41	1.40	
1640	1644	0.00	0.00	1.20		10230	10243	1.77	1.81	1.83	
1705	1711	0.00	0.00	1.50		10254	10260	1.36	1.10	1.42	
1721	1733	1.04	1.52	1.83		10386	10391	1.36	0.00	1.25	
1740	1751	1.05	1.22	1.23		10709	10751	1.12	1.01	2.12	
1759	1766	0.00	1.43	1.77		10946	10978	1.33	1.35	1.74	
1794	1814	1.75	1.69	2.08	+	11029	11040	1.46	1.27	1.69	
1828	1842	1.14	1.11	1.65	+	11066	11080	1.42	1.23	1.43	+
1858	1871	1.26	1.44	1.65		11090	11107	1.72	1.25	1.30	+
1937	1941	1.28	0.00	1.45		11244	11255	1.06	0.00	1.09	+
1961	1971	1.11	1.10	1.27		11369	11387	1.96	1.26	1.79	
2007	2011	0.00	1.09	0.00		11397	11403	1.36	1.11	1.32	
2114	2161	1.94	1.65	1.97		11465	11469	1.20	1.20	1.47	
2290	2315	1.10	1.43	1.51		11510	11519	1.30	1.13	1.59	
2438	2474	1.33	0.00	0.00		11676	11700	0.00	1.29	0.00	
2547	2584	1.15	1.83	1.75		11806	11814	0.00	1.18	1.21	
2598	2613	1.67	1.80	1.86		11824	11832	1.05	0.00	1.42	
2703	2751	2.17	0.00	1.26	+	12086	12098	0.00	0.00	1.48	
2804	2825	1.03	1.39	1.04		12125	12135	0.00	1.44	0.00	
2836	2844	1.07	1.21	1.93		12241	12249	0.00	0.00	1.24	
2866	2875	1.87	1.78	2.28		12263	12274	0.00	1.02	1.52	
2887	2896	1.22	1.32	1.51		12321	12330	1.52	1.83	1.72	
3097	3115	1.81	1.28	1.28		12344	12349	0.00	1.09	1.56	
3144	3154	0.00	1.17	0.00		12367	12384	1.39	1.05	1.63	
3163	3167	0.00	1.16	0.00		12392	12400	1.33	1.26	1.37	
3351	3388	1.84	1.90	2.13		12797	12819	1.32	1.61	1.57	
3404	3424	1.26	1.17	1.51		12835	12846	1.37	1.51	1.58	
3445	3492	1.85	0.00	0.00		12875	12883	1.23	0.00	1.05	
3541	3551	1.02	1.45	1.84		13022	13026	0.00	1.31	0.00	
3557	3569	0.00	1.16	1.56		13057	13061	1.05	0.00	1.45	
4148	4152	1.02	1.24	1.54		13081	13095	1.05	1.05	1.35	+

4175	4184	1.18	1.20	1.21	+	13338	13343	1.14	1.43	1.52	
4192	4219	1.06	1.26	1.68	+	13582	13586	1.54	1.15	1.48	
4333	4377	1.31	1.50	1.63		13612	13617	1.26	1.36	1.76	
4401	4430	1.43	1.10	1.13		13643	13647	0.00	0.00	1.62	
4440	4444	0.00	0.00	1.29		13657	13688	2.02	1.69	2.05	
4510	4514	0.00	0.00	1.41		13778	13809	1.63	1.82	1.14	
4544	4548	1.04	1.10	1.10		13969	13979	1.33	1.67	1.86	
5056	5071	0.00	0.00	1.29	+	13988	14029	1.11	1.23	1.28	
5078	5087	0.00	0.00	1.31	+	14093	14111	1.32	0.00	0.00	
5168	5213	1.59	1.31	1.89		14121	14130	1.14	0.00	0.00	
5460	5466	0.00	1.29	1.46		14449	14455	1.03	0.00	1.62	
5484	5514	1.61	1.56	1.59	+	14479	14484	1.59	1.04	1.44	+
5822	5826	1.32	0.00	0.00		14493	14498	1.50	1.44	1.45	+
6087	6103	1.13	1.27	1.73		14535	14546	1.78	1.44	1.91	+
6240	6245	1.23	1.35	2.03	+	14561	14570	1.25	0.00	1.04	
6353	6369	0.00	0.00	1.73	+	14634	14682	1.61	1.71	1.86	
6404	6409	1.34	0.00	1.08	+	14701	14750	2.01	1.57	1.27	+
6497	6502	1.03	1.30	1.59		14787	14799	1.50	0.00	0.00	+
6539	6550	1.01	0.00	1.20		14832	14840	0.00	1.43	1.71	
6557	6563	1.73	1.55	1.81		14851	14863	1.24	1.05	0.00	
6575	6579	1.04	0.00	1.52		15012	15026	1.07	0.00	1.34	
6609	6635	1.19	0.00	1.26		15041	15046	1.28	1.41	2.04	
6679	6684	1.12	0.00	0.00		15231	15242	1.01	0.00	1.46	
6691	6703	1.61	0.00	1.07		15392	15396	0.00	1.07	1.23	
7132	7139	1.57	1.13	1.66		15463	15467	0.00	1.23	0.00	
7237	7263	1.06	1.10	1.14		15476	15480	0.00	1.02	0.00	
7408	7452	1.29	1.76	0.00	+	15647	15654	1.33	1.31	1.83	
7534	7539	1.22	1.18	0.00		15667	15683	1.02	1.16	1.04	+
7549	7556	1.11	1.15	0.00		16021	16025	1.47	0.00	0.00	+
7791	7795	1.33	1.11	1.55		16075	16080	0.00	0.00	2.06	+
8101	8126	1.49	1.54	1.68		16090	16127	1.18	0.00	2.06	+
8137	8143	0.00	1.40	1.68		16142	16147	0.00	1.01	1.48	+
8175	8183	1.48	0.00	1.42		16164	16186	1.03	1.35	0.00	+
8263	8322	2.32	2.14	1.63	+	16466	16528	6.23	5.85	3.77	
8413	8454	1.55	1.65	1.55							
8529	8581	2.33	1.11	1.11							
8590	8603	0.00	0.00	1.32							
8682	8687	1.29	0.00	1.30							
8723	8783	4.39	3.25	1.89							
8813	8819	1.00	0.00	1.26							
9108	9113	0.00	1.00	1.37	+						

Supplemental Table S6, related to Figure 3: Calculated F-scores for mt-ASFP sites per human embryonic stage. The last column represents adult mt-DGFs that co-localize with mt-ASFP sites.

Human mt-DGF sites >10%			Human mt-DGF sites >10%		
Start	End	% of tested samples	Start	End	% of tested samples
195	335	88.406%	8201	8347	94.203%
400	438	10.145%	8440	8529	89.855%
484	598	91.304%	8546	8607	11.594%
628	687	91.304%	8695	8767	88.406%
832	877	88.406%	8883	8901	36.232%
922	987	76.812%	8923	8976	52.174%
1133	1189	40.580%	9063	9119	98.551%
1229	1253	23.188%	9248	9293	20.290%
1307	1369	65.217%	9414	9509	89.855%
1408	1458	69.565%	9579	9631	50.725%
1475	1556	86.957%	9663	9734	100.000%
1624	1654	73.913%	9741	9764	28.986%
1744	1748	10.145%	9823	9861	17.391%
1785	1857	95.652%	10048	10121	95.652%
1945	2028	78.261%	10166	10252	98.551%
2059	2064	13.043%	10294	10341	98.551%
2088	2155	46.377%	10424	10508	94.203%
2190	2271	17.391%	10609	10694	11.594%
2295	2376	39.130%	10845	10905	88.406%
2404	2416	47.826%	10948	10968	86.957%
2445	2496	89.855%	11046	11121	97.101%
2600	2619	73.913%	11182	11217	40.580%
2660	2674	14.493%	11345	11393	92.754%
2690	2769	95.652%	11432	11465	63.768%
2805	2885	71.014%	11508	11538	40.580%
3089	3202	37.681%	11550	11571	15.942%
3254	3315	73.913%	11581	11603	26.087%
3338	3359	10.145%	11647	11726	72.464%
3416	3450	10.145%	11800	11847	73.913%
3489	3537	53.623%	11917	11966	36.232%
3603	3621	11.594%	11983	11987	24.638%
3694	3768	78.261%	12059	12105	79.710%
3817	3830	28.986%	12144	12196	73.913%
3880	3935	15.942%	12236	12296	60.870%
3980	4032	89.855%	12337	12387	85.507%
4088	4119	86.957%	12431	12449	18.841%
4165	4207	98.551%	12573	12607	89.855%
4259	4323	97.101%	12672	12718	100.000%
4427	4476	23.188%	12781	12795	52.174%
4555	4608	68.116%	12843	12882	71.014%
4633	4686	24.638%	12939	12956	47.826%
4731	4791	85.507%	13012	13038	50.725%
4885	4928	91.304%	13066	13115	94.203%
5049	5106	98.551%	13123	13136	20.290%
5180	5285	79.710%	13155	13199	79.710%

5375	5467	72.464%	13207	13216	18.841%
5480	5577	92.754%	13229	13274	88.406%
5633	5665	66.667%	13337	13346	20.290%
5707	5790	91.304%	13392	13450	98.551%
5832	5921	34.783%	13545	13583	60.870%
5938	5982	68.116%	13701	13734	14.493%
6001	6037	76.812%	13780	13793	75.362%
6099	6135	82.609%	13855	13938	78.261%
6182	6257	95.652%	13944	13966	68.116%
6355	6454	95.652%	14009	14028	62.319%
6480	6502	15.942%	14048	14114	81.159%
6568	6624	18.841%	14181	14213	34.783%
6717	6737	17.391%	14267	14331	82.609%
6782	6800	15.942%	14479	14548	98.551%
6904	6915	13.043%	14586	14618	65.217%
7029	7067	66.667%	14643	14683	33.333%
7148	7179	50.725%	14743	14815	97.101%
7270	7364	94.203%	14987	15001	14.493%
7365	7457	95.652%	15156	15189	47.826%
7498	7570	82.609%	15262	15300	24.638%
7610	7665	26.087%	15398	15467	59.420%
7738	7799	28.986%	15673	15726	94.203%
7827	7836	10.145%	15803	15849	47.826%
7888	7937	55.072%	15899	15917	85.507%
8031	8076	91.304%	15950	16027	94.203%
8109	8112	13.043%	16055	16234	92.754%
8125	8146	14.493%	16274	16340	62.319%

Supplemental Table S7, related to Figure 3: mt-DGF sites in human adult cell lines. mt-DGF sites that were present in more than 10% of the analyzed cell lines (N=70) (according to Blumberg et al. 2018, **Genome Research**).
